# Rebuilding Mitochondrial Homeostasis and Inhibiting Ferroptosis: Therapeutic Mechanisms and Prospects for Spinal Cord Injury

**DOI:** 10.3390/biomedicines13092290

**Published:** 2025-09-18

**Authors:** Qin Wang, Qingqing Qin, Wenqiang Liang, Haoran Guo, Yang Diao, Shengsheng Tian, Xin Wang

**Affiliations:** 1 The First Clinical Medical College, Lanzhou University, Lanzhou 730000, China; 2 Department of Orthopedics, The First Hospital of Lanzhou University, Lanzhou 730000, China

**Keywords:** spinal cord injury, ferroptosis, mitochondrial homeostasis, mechanism, progress

## Abstract

During the pathological process of spinal cord injury (SCI), ferroptosis is closely related to mitochondrial homeostasis. Following the occurrence of SCI, the interruption of local blood supply leads to mitochondrial damage within cells and a reduction in Adenosine triphosphate (ATP) production. This results in the loss of transmembrane ion gradients, causing an influx of Ca^2+^ into the cells, which in turn generates a significant amount of Reactive oxygen species (ROS) and reactive nitrogen species. This leads to severe mitochondrial dysfunction and an imbalance in mitochondrial homeostasis. Ferroptosis is a form of programmed cell death that differs from other types of apoptosis, as it is dependent on the accumulation of iron and lipid peroxides, along with their byproducts. The double bond structures in intracellular polyunsaturated fatty acids (PUFA) are particularly susceptible to attack by ROS, leading to the formation of lipid alkyl free radicals. This accumulation of lipid peroxides within the cells triggers ferroptosis. After SCI, the triggering of ferroptosis is closely associated with the “death triangle”—a core network that catalyzes cell death through the interaction of three factors: local iron overload, collapse of antioxidant defenses, and dysregulation of PUFA metabolism (where PUFA are susceptible to attack by reactive ROS leading to lipid peroxidation). These three elements interact to form a central network driving cell death. In the pathological cascade of SCI, mitochondria serve as both a major source of ROS and a primary target of their attack, playing a crucial role in the initiation and execution of cellular ferroptosis. Mitochondrial homeostasis imbalance is not only a key inducer of the “death triangle” (such as the intensification of lipid peroxidation by mitochondrial ROS), but is also reverse-regulated by the “death triangle” (such as the destruction of mitochondrial structure by lipid peroxidation products). Through the cascade reaction of this triangular network, mitochondrial homeostasis imbalance and the “death triangle” jointly drive the progression of secondary damage. This study aims to synthesize the mechanisms by which various therapeutic approaches mitigate SCI through targeted regulation of mitochondrial homeostasis and inhibition of ferroptosis. Unlike previous research, we integrate the bidirectional regulatory relationship between “mitochondrial homeostasis disruption” and “ferroptosis” in SCI, and emphasize their importance as a synergistic therapeutic target. We not only elaborate in detail how mitochondrial homeostasis—including biogenesis, dynamics, and mitophagy—modulates the initiation and execution of ferroptosis, but also summarize recent strategies that simultaneously target both processes to achieve neuroprotection and functional recovery. Furthermore, this review highlights the translational potential of various treatments in blocking the pathological cascade driven by oxidative stress and lipid peroxidation. These insights provide a novel theoretical framework and propose combinatory therapeutic approaches, thereby laying the groundwork for designing precise and effective comprehensive treatment strategies for SCI in clinical settings.

## 1. Background

SCI is a common neurological condition worldwide, often resulting in impairments in motor function, sensation, and autonomic nervous system function, which can lead to permanent disability. According to recent studies, in 2021, the prevalence and incidence of SCI in China reached 2,766,277 cases and 99,363 cases, respectively, marking increases of 63.27% and 43.27% since 1990. Additionally, an ARIMA forecasting model predicts that by 2036, the age-standardized incidence and prevalence rates of SCI in China will reach 6 cases and 146 cases per 100,000 people, respectively [1]. Due to the fact that SCI not only leads to multi-system dysfunction but also triggers persistent secondary injuries, existing therapies still have significant limitations, in terms of neural function reconstruction and long-term prognosis improvement. Therefore, it is crucial to further elucidate the molecular regulatory networks and cellular cascade response mechanisms involved in SCI. Developing innovative comprehensive treatment strategies that combine neural repair with functional compensation remains a pressing research direction that needs to be addressed.

Maintaining mitochondrial homeostasis is crucial for the normal functioning of cells. Mitochondria are not only central to energy metabolism but also play a role in regulating various physiological processes, including the intracellular redox state, calcium ion balance, and cell signaling. When mitochondrial homeostasis is disrupted, cells may face issues such as energy deficiency, increased oxidative stress, and metabolic disorders, which can lead to cellular dysfunction and death. In recent years, ferroptosis, a novel form of programmed cell death, has garnered significant attention from researchers. The occurrence of ferroptosis is closely linked to the overload of iron ions and lipid peroxidation within cells, with mitochondria playing a critical role in this process. Mitochondrial dysfunction not only promotes the accumulation of iron ions but also exacerbates oxidative stress, thereby driving the onset of ferroptosis. Therefore, investigating the relationship between mitochondrial homeostasis imbalance and ferroptosis is essential for uncovering the mechanisms underlying various diseases and identifying potential therapeutic targets.

Rather than examining mitochondrial homeostasis or ferroptosis in isolation, this review systematically integrates their interplay in SCI. On one hand, it elucidates how disruptions in mitochondrial dynamics and dysregulated mitophagy trigger ferroptosis through mechanisms such as ROS generation and iron metabolism dysregulation. On the other hand, it addresses how lipid peroxidation products generated during ferroptosis exacerbate mitochondrial structural and functional damage. Building on this bidirectional perspective, we summarize synergistic therapeutic strategies that simultaneously target mitochondrial homeostasis—such as enhancing biogenesis and balancing dynamics—and inhibit ferroptosis—for instance, via iron chelation and augmentation of antioxidant systems. These insights provide a new perspective for optimizing the clinical treatment strategies of spinal cord injuries.

## 2. Methodology

To ensure the systematicity and comprehensiveness of this review, we strictly standardized the literature search process. The databases searched included three major English-language databases (PubMed, Web of Science, and Embase).

The search timeframe was restricted to January 2000 through May 2025, aiming to encompass both classic studies and the latest advancements in the field. Search terms included “spinal cord injury,” “ferroptosis,” “mitochondrial homeostasis,” “mitophagy,” “iron overload,” and “GPX4,” among others, combined using the logical operators “AND” and “OR.”

Inclusion criteria were as follows: (1) research focusing on the mutual regulatory mechanisms between ferroptosis and mitochondrial homeostasis following spinal cord injury; (2) inclusion of targeted intervention strategies (e.g., pharmaceuticals, gene regulation, cell transplantation) validated through in vivo or in vitro experiments; (3) original studies or high-quality reviews with complete data and clear conclusions.

The exclusion criteria were as follows: (1) studies solely involving other models (such as brain injury or peripheral nerve injury) that cannot be extrapolated to spinal cord injury; (2) literature with ambiguous mechanistic explanations or duplicate publications; (3) incomplete publications such as abstracts or conference summaries.

After initial screening by title, secondary screening by abstract, and thorough full-text review, 50 core articles were ultimately included, providing a robust literary foundation for the mechanistic analyses and summary of intervention strategies in this review.

## 3. Mitochondrial Dysfunction and Homeostatic Imbalance in SCI

Mitochondrial dysfunction and homeostatic imbalance following SCI are core mechanisms of secondary pathological damage. These processes involve the excessive accumulation of ROS, disruptions in mitochondrial dynamics, and impaired autophagic function, ultimately leading to ferroptosis, hindered axonal regeneration, and irreversible neurological damage. Our previous research has demonstrated that after SCI, damage to the cell membrane results in the dissipation of transmembrane ion gradients. This activates voltage-gated Ca^2+^ channels and causes calcium leakage, further elevating intracellular Ca^2+^ levels. Consequently, this increase enhances the release of the excitatory neurotransmitter glutamate, leading to excitotoxic cell death. Prolonged Ca^2+^ overload can also cause the persistent opening of the mitochondrial permeability transition pore, compromising the barrier function of the inner mitochondrial membrane. Protons continuously enter the mitochondrial matrix, resulting in the loss of the inner membrane potential and uncoupling of oxidative phosphorylation. Due to increased electron leakage, a significant amount of free radicals, such as superoxide (O_2_^−^), is generated, which can further be converted into more destructive hydroxyl radicals (OH^−^) through the Fenton reaction [2]. In fact, the excessive accumulation of ROS can lead to the disruption of mitochondrial homeostasis [3], and impaired mitophagy [4], which further exacerbates the occurrence of ferroptosis [5].

The maintenance of mitochondrial homeostasis is a system-level dynamic equilibrium process, encompassing the synergistic interplay of mitochondrial biogenesis (MB), mitochondrial dynamics, and the regulation of mitophagy. These three components form a functional network through intricate signal crosstalk, all of which are essential for maintaining a healthy and functional mitochondrial network [6].

The normal functioning of mitochondria relies on the dynamic equilibrium of three key processes: mitochondrial biogenesis (MB) supplies new functional units; dynamics (fusion/fission) enable immediate repair through content mixing and segregate damaged components for clearance; and mitophagy is responsible for the ultimate degradation of irreparable components. Following SCI, the energy crisis and Ca^2+^/ROS storm lead to the inhibition of transcription factors such as PGC-1α, obstructing the source of MB. Concurrently, excessive Drp1-mediated fission causes mitochondrial fragmentation, which not only disrupts the networked energy distribution but also prevents damaged mitochondria from being rescued through fusion. Finally, damaged mitochondria accumulate due to disrupted axonal transport, potentially either overwhelming or evading the mitophagic system. This systemic collapse of mitochondrial quality control results in the accumulation of dysfunctional mitochondria, which become major sources of ROS and pro-death signals.

Mitochondrial biogenesis is not a standalone process of quantitative increase, but is closely linked to dynamic remodeling. Peroxisome proliferator-activated receptor-γ coactivator 1α (PGC-1α), acting as the “master regulator” of mitochondrial biogenesis, not only activates the expression of nuclear genes such as mitochondrial transcription factor A (TFAM), but also ensures the rapid integration of newly formed mitochondria into the functional network by regulating the balance between mitochondrial fusion proteins (Mfn1/2) and fission protein (Drp1). Mitochondria are highly dynamic organelles that undergo continuous fission and fusion, a process known as mitochondrial dynamics. This balance is crucial for maintaining a healthy mitochondrial network [7]. Research has found that axonal mitochondria are distributed linearly, with approximately 87% of the mitochondria being stationary. The number of mobile mitochondria gradually decreases from the proximal to the distal end of the axon [8]. Under physiological conditions, mitochondria in axons can move bidirectionally. This transport primarily occurs in two directions: anterograde and retrograde. The purpose of this movement is to allow mitochondria to redistribute themselves in response to the daily physiological demands of the central nervous system, as well as to external stimuli and injuries [9]. Anterograde transport primarily provides neurons with sufficient ATP to ensure their survival. Retrograde transport maintains mitochondrial quality within the axon by dynamically regulating mitochondrial fission and fusion. Damaged mitochondria undergo fission mediated by fission-related proteins such as Drp1 and Fis1, and are then transported back to the cell body via the retrograde transport system, which includes mitochondrial Rho GTPases, motor proteins, and dynein. Once at the cell body, these damaged mitochondria can either be repaired through fusion mediated by mitofusins (Mfn) and optic atrophy protein 1 (OPA1) or degraded via mitophagy. Mitochondrial fission separates damaged mitochondria from healthy ones, while fusion promotes the exchange of components (such as DNA and proteins) to facilitate mutual repair [10]. The dynamic regulatory network formed by these processes coordinates mitochondrial morphology and quantity, ensuring the integrity of mitochondrial function and cell survival under stress conditions [11]. After SCI, the interruption of axonal transport leads to insufficient local energy supply at the damaged site. Concurrently, retrograde transport is disrupted, preventing damaged mitochondria from fusing again to repair themselves and restore the cellular respiratory chain. At this point, simple fission and fusion are no longer sufficient to restore mitochondrial homeostasis in the damaged cells. Consequently, mitophagy is activated as a cellular defense mechanism to eliminate dysfunctional mitochondria that produce elevated levels of ROS [12]. By selectively degrading damaged mitochondria, mitophagy eliminates redundant or dysfunctional mitochondria. This process helps maintain mitochondrial quality and overall cellular health [13].

**Figure 1:**
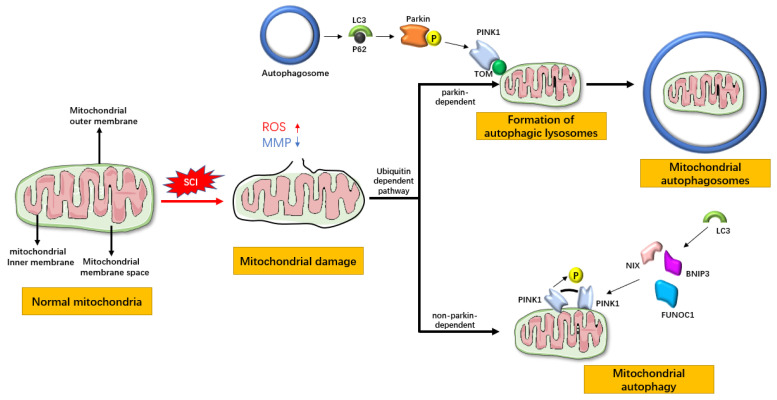
Mitochondrial ubiquitin-dependent autophagy pathway.

Mitochondrial autophagy involves four key steps. First, damaged mitochondria undergo depolarization and lose their membrane potential. Second, an autophagosome forms by enveloping the mitochondria. The third step is the fusion of the autophagosome with a lysosome. Finally, the lysosome degrades the contents of the mitochondria [14]. It primarily relies on the ubiquitin-dependent pathway, which is further divided into Parkin-dependent and non-Parkin-dependent pathways [15]. When mitochondria are damaged, PINK1 binds to TOM and subsequently recruits Parkin from the cytosol, inducing a conformational change in Parkin. This leads to the accumulation of P62, which is bound to LC3, and the formation of autophagolysosomes, thereby completing mitochondrial autophagy through the Parkin-dependent pathway. In the non-ubiquitin-dependent pathway, certain proteins within the mitochondria can directly bind to LC3, allowing mitochondrial autophagy to occur without the need for ubiquitination. Examples of these proteins include NIX, BNIP3, and FUNDC1 [16] (Figure 1). Research by Xu, Jiang, and others has demonstrated that enhancing mitophagy can improve mitochondrial function and serves as a beneficial approach for the treatment of SCI [17,18].

## 4. Ferroptosis in SCI

**Figure 2:**
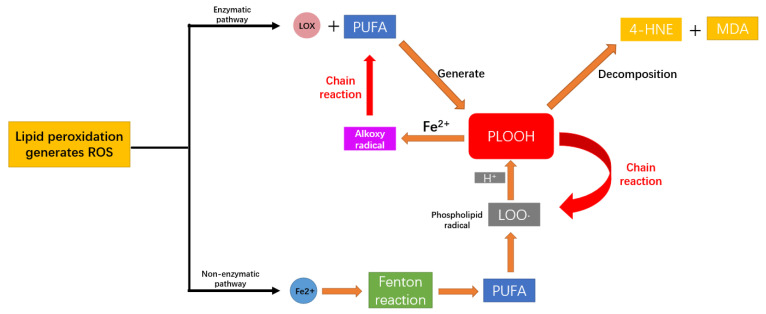
Lipid peroxidation generates ROS.

Ferroptosis is a form of death regulated by autophagy. Research by Yao and colleagues has found that during ferroptosis, neurons undergo excessive mitophagy. This overactive mitophagy not only leads to mitochondrial dysfunction and increased production of ROS but also results in the degradation of ferritin within the mitochondria. Consequently, this leads to an increase in free iron levels within the cells, triggering ferroptosis and creating a vicious cycle [19]. As we discussed earlier, ferroptosis is a process of iron-catalyzed lipid peroxidation, with the most notable feature being the formation of ROS. Within the cell, the production of ROS from lipid peroxidation occurs primarily through two main pathways: enzymatic and non-enzymatic pathways [20]. In the enzymatic pathway, lipoxygenases directly catalyze the formation of phospholipid hydroperoxides (PLOOH) from PUFA. PLOOH can further degrade into 4-hydroxynonenal (4-HNE) and malondialdehyde (MDA), which cross-link with membrane proteins, leading to their inactivation and exacerbating membrane permeability [21]. On the other hand, in the presence of Fe^2+^, PLOOH decomposes into alkoxy radicals, which attack other PUFA and trigger a chain reaction of lipid peroxidation. In the non-enzymatic pathway, excess Fe^2+^ generates a large number of free radicals through the Fenton reaction, which then capture hydrogen ions from PUFA in the plasma membrane, forming phospholipid radicals centered around carbon atoms. These radicals combine with O_2_ to generate PLOOH radicals, which in turn extract hydrogen from another PUFA, resulting in the formation of PLOOH and new phospholipid radicals, thereby also triggering a chain reaction of lipid peroxidation. Both pathways target PUFA, leading to the accumulation of lipid peroxidation products like PLOOH, with Fe^2+^ serving as an essential catalyst in both processes. Ultimately, this results in a chain reaction that rapidly amplifies the damage (Figure 2).

As summarized by Lei and colleagues, lipid peroxides are produced through three mechanisms: iron-catalyzed lipid autoxidation, the esterification and oxygenation of PUFA, and lipid ROS associated with the Fenton reaction. All of these pathways require iron [22]. It has been demonstrated that the occurrence and execution of ferroptosis primarily take place at the intersection of iron, lipid, and amino acid metabolism [23].

## 5. Mitochondrial Homeostasis Regulation Strategies

In fact, the maintenance of mitochondrial homeostasis relies on a highly coordinated and multi-layered dynamic regulatory system that encompasses key processes such as MB, mitochondrial dynamics, and the regulation of mitophagy. These processes are interwoven and collectively respond to changes in both intracellular and extracellular signals, ensuring quality control and functional adaptability of the mitochondrial network. Although research on the regulation of mitochondrial homeostasis is currently limited, existing in vivo and in vitro experiments have already demonstrated exciting results.

MB is one of the core strategies for regulating mitochondrial homeostasis, referring to the process by which cells increase the number of mitochondria and enhance their quality and function. Peroxisome proliferator-activated receptor-gamma coactivator 1-alpha (PGC-1α) serves as the “master regulator” of MB network expression. The activation of PGC-1α has long been considered a therapeutic strategy for SCI, with previous studies demonstrating its neuroprotective effects in both in vivo and in vitro models of SCI [24]. Zhang et al. established a SCI rat model by resecting a 2.0 mm segment of the spinal cord at the T9 vertebra. They developed an injectable in situ gelling hydrogel for the localized delivery of cannabidiol using a novel method based on the polyelectrolyte interactions of sodium carboxymethyl cellulose and chitosan. This gelling hydrogel can be injected into the spinal canal with a 26-gauge syringe and gels within approximately 110 ± 10 s. The results indicated that this composite hydrogel activates the PGC-1α/NRF2 pathway, alleviating mitochondrial dysfunction and cell apoptosis, while also promoting SCI repair more effectively by enhancing mitochondrial biogenesis [25]. LY344864 is a 5-hydroxytryptamine 1F receptor agonist and an effective inducer of MB in multiple organ systems. Epiphani et al. established a SCI model by performing a complete unilateral laminectomy at the T10-T12 thoracic vertebrae in mice, followed by controlled contusion using an Infinite Horizon IH-0400 impactor with a force of 80 kdyn, while ensuring the integrity of the dura mater. To determine the effects of LY344864 on spinal cord MB under physiological conditions, mice received daily intraperitoneal injections of 2.0 mg/kg LY344864 for 21 days, after which the T9–13 region of the spinal cord was isolated for experimental analysis. The results showed that LY344864 treatment significantly mitigated the reduction in PGC-1α as early as 3 days post-SCI, indicating the induction of MB and improvement in mitochondrial homeostasis. Additionally, it promoted the restoration of vascular integrity, enhanced motor function, and reduced lesion volume [26]. In parallel with Epiphani’s research group, Natalie et al. conducted a recent study that revealed another selective 5-HT1F receptor agonist, Lasmiditan, can induce MB in a SCI model. This compound promotes vascular recovery and improves motor function. Furthermore, through cellular experiments, they found that Lasmiditan induces MB and enhances endothelial cell function, potentially via the VE-Cadherin–Akt–FoxO1–claudin-5 signaling axis [27]. Additionally, Natalie et al. established a SCI model using female C57BL/6 mice [27]. They began daily intraperitoneal injections of Formoterol (0.1 mg/kg) one hour after the SCI. Formoterol is a selective and potent β2-adrenergic receptor agonist approved by the U.S. Food and Drug Administration for mitochondrial biogenesis. The results showed that SCI led to a reduction in the expression of mitochondrial proteins in both the injured and surrounding areas, including PGC-1α, Nrf2, TFAM, and ATP Synthase b, while NDUFS1 was completely restored. As early as 3 days post-injury, Formoterol treatment attenuated the decrease in PGC-1α, indicating enhanced MB. Furthermore, the tissue induced by Formoterol was preserved at 3 days post-injury, and there was an increase in the total volume of white and gray matter following treatment. These data suggest that Formoterol may act as a neuroprotective agent, preventing the spread of secondary injury [28]. Finally, they demonstrated that the therapeutic window for Formoterol after SCI is at least 8 h but less than 24 h. Equally important, they observed that this treatment strategy has cross-gender potential, indicating that Formoterol administration has systemic effects on MB [29].

Mitochondria are not static, isolated organelles; rather, they form a highly dynamic and interconnected tubular network, with their morphology, distribution, and interconnectivity undergoing continuous and precisely regulated remodeling. The core mechanism of this dynamic remodeling is known as mitochondrial dynamics, which primarily involves two seemingly opposing yet synergistically unified processes: mitochondrial fusion and fission. Mitochondrial dynamics are far from mere morphological changes; they are essential strategies and foundational frameworks for cells to maintain mitochondrial homeostasis, optimize energy metabolism, execute quality control, respond to environmental stress, and regulate cell fate. The ubiquitin-proteasome system regulates this process by degrading key proteins such as the fusion effectors MFN1 and MFN2. The outer mitochondrial membrane protein PLD6 is a critical regulatory factor downstream of MFN1/2, hydrolyzing cardiolipin to produce the signaling molecule phosphatidic acid. Abnormal levels of PLD6 can lead to excessive mitochondrial fusion or fragmentation [30]. Anat et al. conducted a proteomic analysis to identify mitochondrial substrates of Cullin-RING E3 ligase 2 (CRL2) and its substrate receptor FEM1B. They investigated the interaction mechanism between FEM1B and its substrates, as well as the relationship between FEM1B localization in cells and mitochondria, while also analyzing the role of TOM20 in the mitochondrial localization of FEM1B [31]. The results indicated that TOM20-mediated localization of CRL2-FEM1B on the mitochondrial surface is a core mechanism regulating the degradation of PLD6. This pathway directly influences the balance between mitochondrial fusion and fission by precisely controlling PLD6 protein levels (elevated PLD6 promotes fusion, while reduced PLD6 promotes fission) [31]. Apelin is a peptide produced by the APLN gene and serves as the endogenous ligand for the G protein-coupled receptor APJ. Research has shown that Apelin is expressed in spinal cord neurons and is involved in the repair process following SCI. Maintaining local concentrations of Apelin in the injured area of SCI rats can help preserve the number of neurons at the injury site to some extent [32]. Guo et al. utilized the Hassan Shaker spinal cord transection method and hydrogen peroxide stimulation of PC-12 (highly differentiated) cells to establish in vivo and in vitro models of SCI. They treated the models with PscAAV-hSyn-APLN and XMU-MP-1 injection solutions [33]. The results showed that the selective Mst1 inhibitor XMU-MP-1 directly inhibited Mst1 and reduced Drp1 levels, but did not significantly affect p-JNK. In contrast, Apelin was found to act on both Mst1 and p-JNK to regulate Drp1 and mitochondrial fission. Apelin maintains mitochondrial homeostasis by inhibiting the Mst1–JNK–Drp1 signaling pathway. In both in vivo and in vitro experiments, Apelin protected neurons after SCI and promoted functional recovery by reducing mitochondrial fission, enhancing antioxidant capacity, facilitating the clearance of excess ROS, and decreasing cell apoptosis [33].

Mitochondria are inherently vulnerable organelles within cells due to their continuous exposure to high oxidative stress, metabolic byproducts, and potential risks of genetic mutations. Dysfunctional or structurally damaged mitochondria not only exhibit reduced energy production efficiency but also excessively generate ROS, leak pro-apoptotic factors such as cytochrome c, and disrupt intracellular calcium homeostasis, becoming “dangerous molecules” that threaten cellular health. Therefore, timely identification and removal of these damaged mitochondria are essential for maintaining the overall health of the mitochondrial network and ensuring cellular function and survival. Mitochondrial autophagy, or mitophagy, is a highly specialized form of autophagy that selectively targets, recognizes, encapsulates, and ultimately degrades dysfunctional or redundant mitochondria through the lysosomal pathway, rather than indiscriminately degrading cytoplasmic components. However, in the context of SCI, insufficient mitophagy can lead to the ineffective clearance of damaged mitochondria, resulting in the accumulation of toxic substances, energy metabolism collapse, failure of axonal regeneration, impaired recovery of motor and sensory functions, and exacerbation of secondary injury. Conversely, excessive activation of mitophagy in SCI can lead to the over-removal of normally functioning mitochondria, causing a sharp decline in neuronal ATP synthesis, cellular energy crises, exacerbated acute necrotic cell death, and impaired myelin synthesis, while astrocytes may exacerbate inflammatory responses due to energy depletion. Yao et al. established a mouse model of SCI by compressing the spinal cord at the T10 level and subsequently performed stem cell transplantation [19]. Prior to the compression injury, the mice were randomly divided into three groups: the SCI group (intramedullary injection of a vehicle), the SCI + MSC group (intramedullary injection of untreated MSC suspension), and the SCI + MSC + Cyto D group (intramedullary injection of MSC suspension pre-treated with cytochalasin D). The results showed that mitochondria derived from MSCs could fuse with the damaged neuronal mitochondria and reduce excessive mitophagy during ferroptosis [19]. Yang et al. explored the role of mitophagy regulation in injury-induced spinal cord microglia through animal and cell experiments, as well as single-cell RNA sequencing and metabolomics [7]. Their study confirmed that in an H_2_O_2_-induced SCI model, the activation of the STAT3–FOXO3a signaling pathway inhibits mitochondrial-related gene expression, suppresses superoxide dismutase (SOD) levels, and leads to mitochondrial membrane potential hyperpolarization, decreased mitochondrial quality, increased lipid peroxidation, and reduced ATP levels, resulting in metabolic dysfunction. Furthermore, the pharmacological activator of FOXO3a, TFP, was shown to reduce oxidative stress in SCI mice by modulating mitochondrial activity. Zinc ions were found to promote FOXO3a activation, mediating the expression of mitochondrial-related genes, enhancing mitophagy, restoring mitochondrial stability, improving the spinal cord microenvironment, promoting neuronal survival, and aiding in the recovery of motor function [7]. However, these findings must be viewed critically, as autophagy is a double-edged sword. As previously noted, excessive autophagy may promote ferroptosis by degrading key proteins (such as GPX4) or releasing iron and lipid substrates. Therefore, the focus of future research should be on how to precisely regulate the “degree” and “specificity” of autophagy, thereby directing it toward exerting protective effects. The maintenance of mitochondrial homeostasis relies on the coordinated regulation of biogenesis, dynamic remodeling, and autophagic clearance. Currently, intervention strategies targeting these processes have shown certain potential in SCI models. To systematically compare the characteristics, experimental basis, and limitations of different regulatory approaches, key studies are summarized as follows (Table 1), aiming to provide references for SCI therapies targeting mitochondrial homeostasis.

## 6. Ferroptosis Inhibition Strategy

Ferroptosis is distinct from apoptosis, necroptosis, and autophagy in that it is triggered by the explosive accumulation of iron-dependent lipid peroxidation. Following SCI, local iron overload—resulting from heme degradation and dysregulation of iron transport proteins—combined with the collapse of antioxidant defenses (such as depletion of and inhibition of glutathione peroxidase 4 (GPX4)), as well as an imbalance in PUFA metabolism, creates a “death triangle.” This triangle catalyzes the peroxidative breakdown of membrane phospholipids, resulting in an irreversible loss of cell membrane integrity and, ultimately, cell death. Consequently, current research on strategies to inhibit ferroptosis in SCI models primarily focuses on these three aspects, and some progress has already been made. Primary mechanical injury leads to the rupture of microvessels, causing red blood cells to leak into spinal cord tissue and release large amounts of iron-containing hemoglobin. Subsequently, hemoglobin is gradually broken down into Fe^2+^. Additionally, the acidic environment and reducing agents, such as superoxide anions (O_2_^−^), in the damaged area reduce Fe^3+^ to the more reactive Fe^2+^. This imbalance in iron metabolism results in a dual loss of control characterized by “increased intake” and “blocked output,” triggering a vicious cycle of iron overload. Free Fe^2+^ catalyzes the production of highly reactive hydroxyl radicals through the Fenton reaction, leading to oxidative damage to lipids, proteins, and DNA. Excess Fe^2+^ can also enter the mitochondria via unidirectional transporters, inhibiting respiratory chain complexes and inducing mitochondrial lipid peroxidation. Wang et al. established a SCI model in C57 mice using a high-drop method and administered different concentrations of quercetin [34]. Through immunofluorescence, a ferrous ion colorimetric assay, and Western blot analyses, they found that quercetin reduced intracellular iron accumulation while negatively regulating ferroptosis in oligodendrocyte precursor cells (OPC) through downregulation of the Id2/transferrin pathway, promoting myelin and axon generation. They further investigated whether quercetin prevents ferroptosis in an autophagy-dependent manner. In vitro experiments revealed that quercetin significantly reduced ferrous levels, the LC3II/LC3I ratio, and the number of LC3 puncta in OPC. Pre-treatment with the autophagy inhibitor bafilomycin A1 suppressed quercetin-mediated iron autophagy and ferroptosis, while pre-treatment with the autophagy activator rapamycin reversed the effects of quercetin on ferritin autophagy and ferroptosis in OPC, leading to decreased protein levels of ferritin heavy chain and p62, along with increased levels of LC3II/LC3I and prostaglandin-endoperoxide synthase 2 (PTGS2). In vivo experiments showed that quercetin reduced the levels of NCOA4, PTGS2, and MDA in spinal cord tissue, as well as decreased co-localization of FTH and NCOA4. In summary, quercetin inhibits ferroptosis in OPC by blocking the NCOA4-dependent iron autophagy pathway [34]. Through prolonged continuous experiments, Wang et al. demonstrated that quercetin exerts a dual inhibitory effect, not only by suppressing iron autophagy, which reduces NCOA4 expression and decreases cellular iron release, but also by inhibiting the transferrin pathway, leading to decreased Id2 expression and reduced cellular iron intake, thereby restoring iron homeostasis and inhibiting ferroptosis [34]. Yao et al. established a thoracic SCI model in rats using a modified Allen method [35]. They intervened with intraperitoneal injections of deferoxamine and employed iron concentration assays and Western blot analyses. Their findings revealed that deferoxamine can chelate excess iron, lowering free iron concentrations. Additionally, it inhibits the ferroptosis pathway by upregulating the Xc-/GPX4 axis and suppressing intracellular glutathione (GSH) depletion and lipid ROS. Furthermore, deferoxamine also helps repair mitochondria, protects neurons, and promotes the recovery of motor function [35].

As previously mentioned, following SCI, severe damage to local tissues leads to iron overload and a significant accumulation of ROS. This results in the rapid oxidation of GSH to oxidized glutathione (GSSG), causing a sharp decline in the GSH/GSSG ratio. Consequently, the antioxidant enzyme system, including SOD, GSH, GPX4, and catalase, becomes imbalanced and paralyzed, leading to the accumulation of lipid peroxides that have irreversible effects on cells and tissues. Liu et al. conducted animal experiments to verify that the protective effect of Trimethylpyrazine (TMP) against ferroptosis is mediated through the regulation of the GPX4/ACSL4 axis [36]. Specifically, TMP was found to increase GPX4 levels while simultaneously reducing the levels of acyl-CoA synthetase long-chain family member 4 (ACSL4) [36]. GPX4 is essential in regulating ferroptosis as it converts GSH to its oxidized form. Additionally, it reduces excess lipid peroxides to their corresponding alcohols. Through these redox reactions, GPX4 maintains the balance between oxidation and antioxidant defenses within the cell [37]. ACSL4 is a key isoenzyme involved in the metabolism of PUFA. It converts PUFA, including arachidonic acid, into unsaturated fatty acid phospholipids, thereby increasing the cell’s sensitivity to ferroptosis [38]. Research has shown that inhibiting the expression of GPX4 can enhance the activity of ACSL4, significantly promoting the occurrence of ferroptosis [39]. They established a sham surgery group, an SCI group, a TMP group, an RSL3 group (GPX4 inhibitor), and a TMP + RSL3 group. The results showed that, compared to the other control groups, the TMP group significantly reduced intracellular Fe^2+^, ROS, and MDA concentrations while increasing GSH and SOD levels. These findings indicate that TMP regulates GPX4 and ACSL4 to maintain redox balance within the cells, thereby alleviating neuronal ferroptosis and providing protective effects in neurons surrounding the injury site. Additionally, zinc is closely related to neuronal growth, maturation, and energy metabolism [40]. Relevant studies have shown that an appropriate amount of zinc can exhibit antioxidant, anti-apoptotic, and immune-regulatory effects [41]. Research by Ge et al. demonstrated that zinc reverses behavioral and structural changes following SCI [42]. It increases the expression of NRF2 and HO-1, leading to elevated levels of GPX4, SOD, and GSH, while reducing levels of lipid peroxides, MDA, and ROS. Zinc also helps rescue damaged mitochondria and effectively reduces levels of SCI and inflammatory factors. The NRF2 inhibitor Brusatol was found to reverse the effects of zinc. In their study, the researchers randomly divided mice into four groups: a sham surgery group, an SCI group, an SCI + ZnG injection group, and an SCI + ZnG + Brusatol group. The results indicated that the expression of NRF2 in the SCI + ZnG group was significantly higher than in the SCI group, and the expression level of HO-1 was also significantly greater than in the SCI + inhibitor group. Immunofluorescence further revealed that the expression of NRF2 and HO-1 in spinal cord neurons treated with zinc was significantly increased compared to those treated with the SCI + vehicle. Overall, these findings provide strong evidence that ZnG inhibits the ferroptosis pathway by upregulating the expression of NRF2, HO-1, and GPX4, while suppressing lipid peroxidation [42].

When SCI leads to the collapse of the antioxidant system (characterized by GPX4 inactivation, GSH depletion, and inhibition of the Nrf2 pathway), the lipid metabolic environment deteriorates and gradually becomes imbalanced. PUFA, due to their multiple double bonds and the presence of hydrogen atoms on the methylene groups between these double bonds, are easily attacked by free radicals, triggering chain reactions. Furthermore, because PUFA constitute a significant proportion of neuronal membrane phospholipids, their high sensitivity to oxidation makes them core targets of oxidative stress. This process involves reactions with molecular oxygen, which can initiate further chain reactions. Importantly, since this process does not require the mediation of biological enzymes, the damage can rapidly spread to adjacent healthy cells [43]. This leads to a significant occurrence of ferroptosis in spinal cord cells. Adiponectin (APN) is an adipocyte-derived adipokine that plays a crucial role in regulating glucose uptake and lipid metabolism [44], APN regulates various physiological processes through its receptors, namely AdipoR1, AdipoR2, and T-cadherin. In the central nervous system of both humans and rodents, AdipoRs are expressed in neurons, endothelial cells, and astrocytes [45], The neuroprotective effects of APN have been demonstrated in a wide range of studies [46]. Ge et al. found through clinical data that serum APN levels are correlated with long-term prognosis in patients with traumatic brain injury (TBI) [47]. Subsequent animal studies indicated that genetic blockade of the APN–AdipoR1 pathway significantly exacerbated ferroptosis following TBI, while activation of AdipoR1 markedly reduced ferroptosis and improved neurological outcomes. Non-targeted lipidomic analysis revealed that AdipoRon inhibited the production of various lipids, including PUFA such as arachidonic acid, docosahexaenoic acid, and linoleic acid, which were increased after TBI. CCK8 assays demonstrated that AdipoRon significantly reduced the production of MDA and 4-HNE, as well as the elevated expression of NOX2 and COX2. In summary, APN prevents ferroptosis by activating AMP-activated protein kinase and inhibiting acetyl-CoA carboxylase-1-mediated fatty acid synthesis [47]. Shen et al.’s in vivo experiments demonstrated that Celastrol, also known as Tripterygium wilfordii, is a natural pentacyclic triterpene isolated from the plant [48]. Its neuroprotective effects in the central nervous system are gradually being demonstrated. In a study using a rat model of SCI created through laminectomy and heavy weight drop, the experimental group received intraperitoneal injections of different concentrations of Celastrol. The results showed that Celastrol promoted the recovery of spinal cord tissue and motor function in SCI rats. Further in vitro and in vivo studies indicated that Celastrol significantly inhibited ferroptosis in neurons and OPC while reducing the accumulation of ROS. In summary, Celastrol can reduce lipid peroxidation and alleviate ferroptosis in neurons and OPC by upregulating the NRF2–xCT–GPX4 axis [49]. Additionally, research by Li et al. demonstrated that dihydroorotate dehydrogenase is a potent inhibitor of ferroptosis [50]. It has been shown to inhibit the activation of ferroptosis-related molecules and reduce the production of lipid peroxides and mitochondrial damage. The mechanism of action involves the ability of dihydroorotate dehydrogenase to reduce ferroptosis in neurons following SCI by inhibiting the P53/ALOX15 signaling pathway, thereby alleviating the effects of SCI [50]. While these inhibitors have shown great potential in preclinical models, a critical challenge lies in their off-target effects. For instance, iron chelators may induce systemic anemia, and antioxidants could disrupt normal redox signaling. Therefore, the development of cell- or tissue-specific targeted delivery systems will be pivotal to advancing these therapies toward clinical application. To systematically summarize the current intervention strategies targeting ferroptosis and their associations with the “death triangle” (iron overload, collapse of antioxidant defense, and imbalance in PUFA metabolism), as well as to clarify the characteristics and limitations of different approaches in SCI models, the comparative evidence is compiled as follows (Table 2).

## 7. Biomarkers and Monitoring Significance of Mitochondrial Function and Ferroptosis

Dynamic monitoring of mitochondrial function and ferroptosis is critical for evaluating the pathological progression of SCI and the efficacy of interventions, with the identification of specific biomarkers providing quantifiable metrics for this process. For monitoring mitochondrial function, intracellular ATP levels directly reflect mitochondrial respiratory chain activity; an elevated lactate/pyruvate ratio indicates impaired oxidative phosphorylation; mitochondria-derived superoxide anions (O_2_^−^) can be tracked in real time using targeted probes; and mtDNA release, along with abnormal expression of proteins such as TFAM and Tom20, respectively, indicate changes in mitochondrial membrane permeability and disruption of structural integrity. In the context of ferroptosis, levels of free Fe^2+^, transferrin saturation, and changes in serum ferritin reflect iron metabolic imbalance; lipid peroxidation end products (MDA, 4-HNE) and specific oxidized phospholipids (e.g., PE-OOH) serve as direct markers of lipid damage; a decreased GSH/GSSG ratio and reduced GPX4 activity signal the collapse of the antioxidant system.

Combined detection of these biomarkers can form a dynamic assessment network linking “mitochondrial function-ferroptosis.” For example, a sharp early drop in ATP accompanied by elevated Fe^2+^ and 4-HNE after SCI indicates the need for urgent combined intervention; during the recovery phase, Rebound GPX4 activity and stable TFAM levels predict a favorable therapeutic response. Future development of minimally invasive detection technologies (e.g., rapid detection of specific biomarkers in cerebrospinal fluid) will enable real-time regulation of intervention strategies, providing crucial support for precision therapy in SCI.

## 8. Summary and Prospect

In fact, targeting the regulation of mitochondrial homeostasis and inhibiting ferroptosis represents a promising new therapeutic strategy. These two approaches are not isolated; the imbalance of mitochondrial homeostasis is a key trigger for ferroptosis, and ferroptosis can further exacerbate mitochondrial damage, creating a vicious cycle. Therefore, future research should delve deeper into the mechanisms of interaction between these two processes and focus on developing integrative therapies. Key issues to be focused on include how to achieve precise regulation of mitophagy to avoid insufficient or excessive activation while adapting to the needs of different cell types; whether combined therapies, such as mitochondrial biogenesis enhancers and ferroptosis inhibitors, exert synergistic effects; how to optimize their administration timing and dosage compatibility to avoid potential conflicts; and how gene therapies (e.g., targeted regulation of PGC-1α, ACSL4) and mitochondrial transplantation techniques can be combined with existing intervention strategies.

Despite challenges in clinical translation, including drug delivery efficiency, long-term safety validation, and individual variability, research focused on mitochondrial homeostasis and ferroptosis undoubtedly contributes to overcoming the limitations of traditional treatments. We have reason to believe that this therapeutic approach will bring new hope and attention to the treatment of SCI.

## Figures and Tables

**Table 1:** Comparison of experimental evidence for mitochondrial homeostasis regulation strategies in spinal cord injury.

Intervention Type	Intervention/Target	Model Type	Key Outcomes	Limitations
Mitochondrial Biogenesis (MB) Regulation	Cannabidiol-loaded in situ gelling hydrogel	In vivo: Rat model with T9 spinal cord segment resection	Activates PGC-1α/NRF2 pathway, enhances MB, alleviates mitochondrial dysfunction and apoptosis, and promotes SCI repair	Hydrogel degradation rate and long-term safety not clarified; no evaluation of effects on chronic-phase nerve regeneration
	LY344864 (5-HT1F receptor agonist)	In vivo: Mouse model with T10–12 thoracic contusion (80 kdyn force)	Attenuates PGC-1α reduction as early as 3 days post-injury, improves mitochondrial homeostasis, promotes vascular repair, motor function recovery, and lesion size reduction	Only verified within 21 days; no data on universality across different spinal segment injuries
	Lasmiditan (5-HT1FR agonist)	In vivo: SCI model mice; In vitro: Endothelial cells	Induces MB, enhances endothelial cell function via the VE-Cadherin–Akt–FoxO1–claudin-5 axis, and improves vascular recovery and motor function	Endothelial cell mechanisms not validated in spinal neurons; lacks dose-dependent data
	Formoterol (β2-adrenergic receptor agonist)	In vivo: SCI model in female C57BL/6 mice	Restores expression of mitochondrial proteins (e.g., PGC-1α, Nrf2) 3 days post-injury, increases white/gray matter volume, with a treatment window of at least 8 h and cross-sex potential	Efficacy details in male models insufficiently elaborated; no analysis of long-term effects on organs like the heart
Mitochondrial Dynamics Regulation	CRL2–FEM1B–PLD6 pathway	In vitro: Cell experiments (focusing on mitochondrial localization and degradation mechanisms)	TOM20 mediates CRL2-FEM1B localization to mitochondria, balancing mitochondrial fusion/fission by regulating PLD6 levels	Not validated in in vivo SCI models; lacks direct evaluation of neuronal function
	Apelin (targeting Mst1–JNK–Drp1 pathway)	In vitro: H_2_O_2_-stimulated PC-12 cells; In vivo: Rat spinal cord transection model	Inhibits mitochondrial fission, enhances antioxidant capacity, clears excess ROS, reduces apoptosis, protects neurons, and promotes functional recovery post-SCI	Interactions with fusion-related proteins (e.g., Mfn1/2) not clarified; insufficient dose optimization for long-term administration
Mitophagy Regulation	Mesenchymal Stem Cell (MSC) transplantation	In vivo: Mouse model with T10 spinal cord crush injury	MSC-derived mitochondria fuse with damaged neuronal mitochondria, reducing excessive mitophagy associated with ferroptosis	Immunological rejection risks of MSC transplantation unassessed; functional integrity of fused mitochondria not confirmed
	Zinc ions/TFP (FOXO3a activator)	In vitro: H_2_O_2_-induced SCI cell model; In vivo: SCI mouse model	Activates FOXO3a, enhances mitophagy, restores mitochondrial stability, improves spinal microenvironment, and promotes motor function recovery	Synergistic effects of zinc ions and TFP not explored; no analysis of effects on astrocyte autophagy

**Table 2:** Comparison of main intervention strategies targeting ferroptosis and their mechanisms in spinal cord injury.

Intervention	Model Type	Mechanism Targeting the “Death Triangle”	Key Outcomes	Limitations
Quercetin	In vitro: OPCs In vivo: C57 mice with T10 weight-drop SCI	Inhibits iron overload (blocks NCOA4-mediated ferritinophagy + downregulates Id2/transferrin pathway, reducing iron release and uptake)	Reduces intracellular Fe^2+^, NCOA4, and PTGS2 levels; decreases MDA accumulation; promotes myelin and axon formation	In vitro studies only focused on OPCs, excluding neurons/astrocytes; toxicity data for high in vivo doses (>100 mg/kg) are lacking
Deferoxamine	In vitro: Neurons In vivo: Rats with T10 modified Allen’s contusion	Inhibits iron overload (chelates free Fe^2+^) + enhances antioxidant defense (upregulates Xc^−^/GPX4 axis, reducing GSH depletion)	Decreases free iron concentration and lipid ROS; repairs mitochondrial function; improves motor function	Long-term use may cause systemic iron deficiency; no evaluation of axonal regeneration in the chronic phase (>4 weeks)
Tetramethylpyrazine (TMP)	In vitro: Neurons In vivo: Rats with laminectomy-induced contusion	Enhances antioxidant defense (upregulates GPX4) + regulates PUFA metabolism (downregulates ACSL4, reducing conversion of PUFA to oxidizable phospholipids)	Increases GSH and SOD levels; decreases Fe^2+^, ROS, and MDA; inhibits neuronal ferroptosis	In vitro effects on glial cells not verified; in vivo lacks stratified data for different injury severities (e.g., complete/incomplete SCI)
Zinc (ZnG)	In vitro: Neurons + astrocytes In vivo: Mice with T10 contusion	Enhances antioxidant defense (activates NRF2/HO-1 pathway, upregulates GPX4 and SOD)	Reduces lipid peroxides, MDA, and ROS; rescues mitochondrial function; decreases inflammatory factors	No analysis of gender differences (zinc metabolism is gender-specific); high doses (>20 mg/kg) may induce neuronal apoptosis
Adiponectin (APN)	In vitro: Neurons In vivo: TBI mouse model (extrapolatable to SCI)	Regulates PUFA metabolism (inhibits production of PUFA such as arachidonic acid) + enhances antioxidant defense (activates AMPK pathway)	Reduces MDA, 4-HNE, and NOX2/COX2 expression; alleviates ferroptosis-related lipid peroxidation	Lacks direct data in SCI models; unclear effects on spinal cord-specific cells (e.g., oligodendrocytes)
Celastrol	In vitro: Neurons + OPCs In vivo: Rats with T10 weight-drop contusion	Enhances antioxidant defense (upregulates NRF2–xCT–GPX4 axis)	Reduces ROS accumulation; inhibits ferroptosis in neurons and OPCs; promotes motor function recovery	In vitro dose–effect relationship not clarified; long-term in vivo toxicity (e.g., liver injury) needs evaluation
Dihydroorotate dehydrogenase	In vitro: Neurons In vivo: SCI rat model	Enhances antioxidant defense (inhibits P53/ALOX15 pathway, reducing lipid peroxides)	Decreases activity of ferroptosis-related molecules; reduces mitochondrial damage	No clear direct association with the “death triangle” in its mechanism; clinical translation potential remains to be verified

## Abbreviations

The following abbreviations are used in this manuscript: Spinal cord injury (SCI), Adenosine triphosphate (ATP), Reactive oxygen species (ROS), Polyunsaturated fatty acids (PUFA), Mitochondrial biogenesis (MB), Phospholipid hydroperoxides (PLOOH), 4-Hydroxynonenal (4-HNE), Malondialdehyde (MDA), Peroxisome proliferator-activated receptor-γ coactivator-1α (PGC-1α), Oligodendrocyte precursor cells (OPC), Reduced glutathione (GSH), Oxidized glutathione (GSSG), Superoxide dismutase (SOD), Glutathione peroxidase 4 (GPX4), Acyl-CoA synthetase long-chain family member 4 (ACSL4), Trimethylpyrazine (TMP), Adiponectin (APN), Traumatic brain injury (TBI).
